# Hospitalizations for Respiratory Disease Among Unaccompanied Children from Central America — Multiple States, June–July 2014

**Published:** 2014-08-15

**Authors:** Edith N. Nyangoma, Carmen Sofia Arriola, Jose Hagan, Christina Socias, Sara Tomczyk, Louise Francois Watkins, Matthew Westercamp, Curi Kim

**Affiliations:** 1 Epidemic Intelligence Service, CDC; 2 Division of Global Migration and Quarantine, National Center for Emerging and Zoonotic Infectious Diseases, CDC; 3 Influenza Division, National Center for Immunization and Respiratory Diseases, CDC; 4 Global Immunization Division, Center for Global Health; 5 Division of Safety Research, National Institute for Occupational Safety and Health, CDC; 6 Division of Bacterial Diseases, National Center for Immunization and Respiratory Diseases, CDC; 7 Office of Refugee Resettlement, Administration for Children and Families

During October 2013–June 2014, approximately 54,000 unaccompanied children, mostly from the Central American countries of El Salvador, Guatemala, and Honduras, were identified attempting entry into the United States from Mexico, exceeding numbers reported in previous years (1). Once identified in the United States, U.S. Customs and Border Protection, an agency of the U.S. Department of Homeland Security, processes the unaccompanied children and transfers them to the Office of Refugee Resettlement (ORR), an office of the Administration for Children and Families, U.S. Department of Health and Human Services. ORR cares for the children in shelters until they can be released to a sponsor, typically a parent or relative, who can care for the child while their immigration case is processed. In June 2014, in response to the increased number of unaccompanied children, U.S. Customs and Border Protection expanded operations to accommodate children at a processing center in Nogales, Arizona. ORR, together with the U.S. Department of Defense, opened additional large temporary shelters for the children at Lackland Air Force Base, Texas; U.S. Army Garrison Ft. Sill, Oklahoma; and Naval Base Ventura County, California.

On July 10, 2014, CDC was informed by the California Department of Public Health and ORR about four unaccompanied male children aged 14–16 years with respiratory illnesses at Naval Base Ventura County, three of whom were hospitalized with pneumonia. Among the three patients with pneumonia, two were bacteremic with *Streptococcus pneumoniae*, ultimately determined to be serotype 5, one of whom also had laboratory-confirmed influenza B virus by polymerase chain reaction (PCR). The fourth patient, without pneumonia, had PCR-confirmed influenza A(H1N1)pdm09. Pneumococcal bacteremia is uncommon among U.S. adolescents, particularly serotype 5, with only three such cases identified in the past 10 years by CDC (2). In addition, influenza activity in the United States is typically lowest in the middle of summer, and Ventura County had no reports of an unusual increase in influenza activity in the community at the time. ORR asked CDC to investigate the scope of this apparent outbreak and implement measures to interrupt transmission.

During July 6–19, 2014, CDC was informed of other clusters of hospitalized children with respiratory disease, increasing the total to 16 cases. The cases were from Naval Base Ventura County (eight cases), Ft. Sill (three), Lackland Air Force Base (two), a standard ORR shelter near Houston, Texas (two), and the Nogales processing center (one). Cases were in persons aged 14–17 years. Diagnoses included laboratory-confirmed pneumococcal pneumonia with laboratory-confirmed influenza (three cases) and without laboratory-confirmed influenza (four cases), influenza pneumonia (one case), and pneumonia with no identified etiology (eight cases). Five patients experienced septic shock requiring intensive care. No case was fatal. All six cases for which pneumococcal isolates were available were identified as serotype 5, a serotype included in 13-valent pneumococcal conjugate vaccine (PCV13) (Prevnar-13, Pfizer). Of the 16 patients identified in this cluster, 11 were tested for influenza viruses; four (36%) were positive (two for influenza A[H1N1]pdm09, one for influenza B, and one for influenza A by rapid test).

Because of the concern that unaccompanied children were at increased risk for influenza and pneumococcal pneumonia in this outbreak setting and the clinically important interaction between influenza and pneumococcal infections (3), CDC recommended that all children residing in temporary or standard ORR shelters receive influenza vaccine and PCV13 in addition to routinely recommended vaccines. Approximately 2,000 children in four affected shelters were vaccinated during July 18–30 with PCV13 and with Food and Drug Administration–approved extended expiration date–specific lots of 2013–14 seasonal influenza vaccine, which includes influenza A(H1N1)pdm09 and influenza B viruses. The shelters reported no serious adverse events.

Although some countries in Central America recommend influenza vaccination for young children, school-aged children generally are not targeted for vaccination (4). Routine annual influenza vaccination is recommended for all persons in the United States aged ≥6 months (5). Because influenza activity was identified among the unaccompanied children, this outbreak underscores the importance of providing routine influenza vaccinations to this population.

PCV13 is routinely given in the United States at age 2–59 months. It is recommended for the older unaccompanied children because of the unexpected number of pneumococcal pneumonia cases occurring in the context of crowded conditions that likely facilitate spread of respiratory agents and because the risk for serious pneumococcal disease is increased with the circulation of influenza viruses.

Efforts by state and local public health departments were crucial in identifying disease clusters among the children, assisting in investigating the clusters, and supporting immunization activities, highlighting the critical role of state and local health departments working with federal agencies in detecting and responding to outbreaks. Additional information about the ongoing humanitarian and public health response is available at http://emergency.cdc.gov/children/unaccompanied/index.asp.

## References

[1] US Customs and Border Protection (2014). Southwest border unaccompanied alien children.

[2] CDC (2014). Active Bacterial Core surveillance (ABCs).

[3] Brundage JF (2006). Interactions between influenza and bacterial respiratory pathogens: implications for pandemic preparedness. Lancet Infect Dis.

[4] Ropero-Álvarez AM, Kurtis HJ, Danovaro-Holliday MC (2009). Expansion of seasonal influenza vaccination in the Americas. BMC Public Health.

[5] CDC (2013). Summary recommendations: prevention and control of influenza with vaccines: recommendations of the Advisory Committee on Immunization Practices (ACIP)—United States, 2013–14.

